# Climate-Driven Effects of Fire on Winter Habitat for Caribou in the Alaskan-Yukon Arctic

**DOI:** 10.1371/journal.pone.0100588

**Published:** 2014-07-03

**Authors:** David D. Gustine, Todd J. Brinkman, Michael A. Lindgren, Jennifer I. Schmidt, T. Scott Rupp, Layne G. Adams

**Affiliations:** 1 U. S. Geological Survey, Alaska Science Center, Anchorage, Alaska, United States of America; 2 Scenarios Network for Alaska and Arctic Planning, University of Alaska Fairbanks, Fairbanks, Alaska, United States of America; DOE Pacific Northwest National Laboratory, United States of America

## Abstract

Climatic warming has direct implications for fire-dominated disturbance patterns in northern ecosystems. A transforming wildfire regime is altering plant composition and successional patterns, thus affecting the distribution and potentially the abundance of large herbivores. Caribou (*Rangifer tarandus*) are an important subsistence resource for communities throughout the north and a species that depends on terrestrial lichen in late-successional forests and tundra systems. Projected increases in area burned and reductions in stand ages may reduce lichen availability within caribou winter ranges. Sufficient reductions in lichen abundance could alter the capacity of these areas to support caribou populations. To assess the potential role of a changing fire regime on winter habitat for caribou, we used a simulation modeling platform, two global circulation models (GCMs), and a moderate emissions scenario to project annual fire characteristics and the resulting abundance of lichen-producing vegetation types (i.e., spruce forests and tundra >60 years old) across a modeling domain that encompassed the winter ranges of the Central Arctic and Porcupine caribou herds in the Alaskan-Yukon Arctic. Fires were less numerous and smaller in tundra compared to spruce habitats throughout the 90-year projection for both GCMs. Given the more likely climate trajectory, we projected that the Porcupine caribou herd, which winters primarily in the boreal forest, could be expected to experience a greater reduction in lichen-producing winter habitats (−21%) than the Central Arctic herd that wintered primarily in the arctic tundra (−11%). Our results suggest that caribou herds wintering in boreal forest will undergo fire-driven reductions in lichen-producing habitats that will, at a minimum, alter their distribution. Range shifts of caribou resulting from fire-driven changes to winter habitat may diminish access to caribou for rural communities that reside in fire-prone areas.

## Introduction

Recent and projected climatic warming has direct implications for fire-dominated disturbance regimes [1], particularly at high latitudes where warming has been amplified [2]. Evidence suggests strong linkages among the increased temperatures and altered precipitation patterns associated with climate warming and increases in wildfire frequency, severity, and area burned in boreal forests of North America [3]–[6]. For example, half of the largest fire years in Alaska's 60-year record have occurred since 1990 and two of the three most extensive wildfire seasons have happened in the last decade [7].

A transforming wildfire regime is affecting ecosystem structure and function in the north by altering plant composition and successional patterns [8]. These shifts in vegetation alter the distribution and abundance of northern herbivores in different ways [9]. Wildfire may benefit some species, such as moose (*Alces alces*), by increasing early-successional habitats [10], [11]. However, wildfire may have negative effects on species dependent on late successional habitats, such as caribou (*Rangifer tarandus*) [11]–[15].

Understanding impacts of a changing wildfire regime on caribou is important for social and ecological reasons. Culturally, barren-ground or migratory tundra caribou, the ecotype occurring in the North American Arctic [13], [16], constitute the most important terrestrial resource for subsistence hunters throughout the region, with many indigenous groups identifying themselves as “caribou people” [17], [18]. Ecologically, migratory tundra caribou are distributed across areas experiencing the most evident and unprecedented changes resulting from climate warming [19]. Of all the climate-driven factors affecting shifts in vegetation types at northern latitudes, fire has the most potential to rapidly alter the composition and distribution of plant communities that caribou use in boreal forest and tundra systems [11], [12], [20]. Gaining insights to how fire-related disturbance may affect caribou distribution on the landscape will aid in understanding challenges facing people who rely on caribou as a crucial subsistence resource.

Migratory tundra caribou that occur throughout the Alaskan and Canadian Arctic are highly gregarious, occur at high localized densities, aggregate on calving grounds to bear young, undergo long seasonal migrations, and have very large annual ranges. Typically, these caribou migrate to the arctic tundra for the growing season and winter in the boreal forest and the forest-tundra interface [13], [21], where they subsist primarily on the terrestrial lichens (*Cladonia* spp.) [22]–[25] that occur in late-succession habitats [12], [26]. Indeed, the availability of high energy forage lichens throughout the winter reduces the reliance of caribou on body stores [27] that are vital to survival and reproduction [28]. The loss of lichen-rich vegetative associations, such as lichen-bearing spruce (*Picea* spp.) forests and tundra communities, could alter the distribution of caribou in winter [29], [30] and possibly influence population dynamics [11], [31], [32].

Fire exerts a profound effect on the abundance of lichen in boreal forest and tundra ecosystems [30], [33]. In Alaskan forests, the past low-frequency fire regime created large patches of relatively old-aged stands. However, the frequency of large fires and the total area burned annually has been increasing [34]–[36]; this trend is expected to continue well into the 21^st^ century [11]. In young, recently-burned vegetation, forage lichens are largely absent, while lichen abundance is highest in older conifer stands [12], [30], [37]. On the scale of centuries, large fires may enhance nutrient cycling and promote habitat diversity for caribou [30], but in the shorter term, lichen abundance is greatly reduced and remains low for decades [32]. In east-central Alaska, caribou strongly selected spruce stands that were >80 years old while stands <60 years old were rarely used and lichen biomass and recovery post-fire were strongly correlated with these patterns of use [12], [38].

Although less is understood about fire history in tundra communities, the effect of fire on forage lichens is similar to that of the boreal forest. Fires in the tundra biome were rare in the past 2,000–5,000 years [6], [20], but Paleo-climate records show marked increases in fire frequencies during warming periods that coincided with expansion of shrubs into tundra communities [20], [39]. Changes in hydrology and temperature regimes [40] will likely increase the drying of tussock tundra throughout the growing season and increase the frequency of fires [6]. Although several factors, such as burn severity and edaphic characteristics, can influence succession following a tundra fire, burns in the tundra are typically recolonized by graminoids and shrubs [41], [42]. The presence of these vascular plants, especially deciduous shrubs, may further increase fire frequency [20] and thereby preclude forage lichens from recolonizing [42], [43]. Simulations in northwest Alaska suggest that further increases in temperature could double the total area burned per decade in tundra systems [11], [44]. As in the boreal forest, tundra fires affect the distribution of caribou in winter for decades: caribou typically avoided burns <60 years old in northwest Alaska [14], [29].

**Figure 1:**
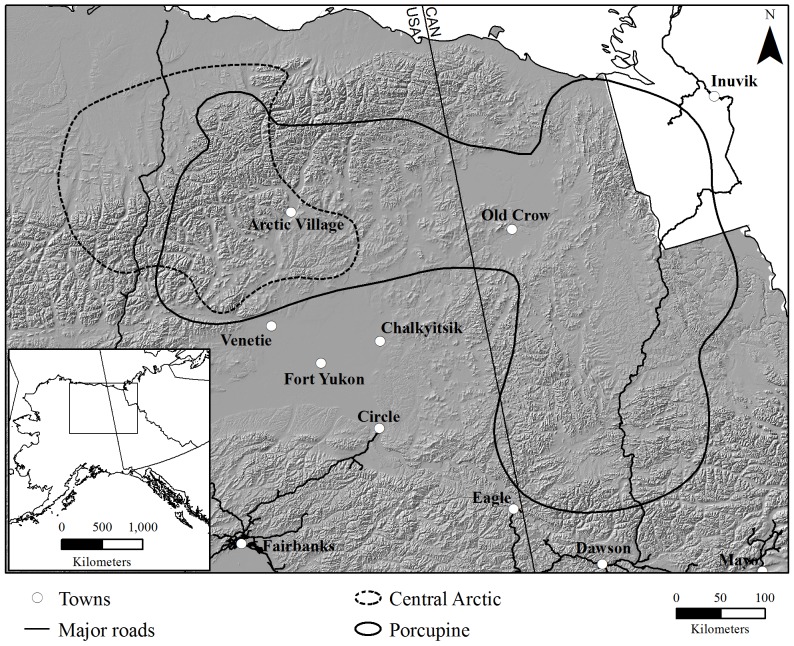
Simulation domain and winter ranges of the Central Arctic and Porcupine caribou herds, Alaska and Yukon.

To explore the effects of the climate-fire dynamic on the availability of lichen-producing winter habitats (hereafter to referred to as winter habitat) for migratory tundra caribou through 2100, we used an established simulation modeling platform [Alaskan Frame-based Ecosystem Code (ALFRESCO)] [45], and employed two global circulation models (GCMs) that defined the range of plausible climate projections for northwestern North America along with a moderate emissions scenario, to project fire regime characteristics, the abundance of winter habitat (tundra and spruce forest stands >60 years old), and the relative flammability (defined below) of the winter ranges of the Central Arctic and Porcupine caribou herds in the Alaskan-Yukon Arctic (Fig. 1). These adjacent herds represent the two wintering behaviors observed in migratory tundra caribou populations: the Central Arctic herd primarily uses Arctic and montane tundra habitats whereas the Porcupine herd typically winters in boreal forest habitats at or south of latitudinal treeline. The first analysis of this type was conducted on the Nelchina caribou herd in Interior Alaska [15] where basic hypotheses on the response of vegetation to a changing fire regime were tested. Contrary to previous work [11], we used markedly different current and projected temperature and precipitation regimes than in western Alaska. Thus, we used different vegetative inputs and contrasted the climate-mediated changes to winter habitat of two herds in a region influenced by the continental-montane-arctic climates. Therefore, our research represents modeling advancements that provide state-of-the-science scenarios of biophysical change, expanding the relevance and application of our findings to management, research, and conservation as well as subsistence-based communities throughout northern North America.

## Materials and Methods

### Study area and caribou herds

The simulation domain comprised 570,112 km^2^ of eastern Alaskan and western Canadian boreal and arctic ecosystems, encompassing the winter ranges of the Central Arctic and Porcupine caribou herds (named after the Porcupine River; Fig. 1). There was a large physiographic gradient (0–3,200 m) that included boreal deciduous and coniferous forests, expansive wetlands, and arctic and alpine tundra. Since the mid-1970s when regular monitoring of these herds began [46], the Central Arctic herd has increased markedly from 5,000 to 70,000, whereas the Porcupine herd increased from 100,000 to 178,000 in the late 1980s and now numbers around 169,000 [47]. Winter range extents for each herd were based on over 30 years of radiotelemetry studies. Specifically, the Central Arctic winter range was based on a combination of telemetry data and expert opinion [48], whereas the winter range of the Porcupine herd was derived via a 90% adaptive kernel algorithm applied to available telemetry data (K. Poole, Aurora Wildlife Research, unpublished analyses). The historic winter ranges of the Central Arctic (60,175 km^2^) and Porcupine (176,540 km^2^) caribou herds span east from the Colville River in north-central Alaska to the Northwest Territories and north from the Yukon River to the Beaufort Sea (Fig. 1). The Central Arctic herd primarily winters in tundra habitats that span the northern and southern slopes of the Brooks Range, while the Porcupine herd winters predominantly in the northern interior boreal regions of Alaska and Yukon (Fig. 1). Detailed descriptions of the ranges for each herd have been provided elsewhere [23], [49], [50].

### Fire

We used the “raster” package [51] in the R statistical language version 2.15.2 (R Core Team, http://www.R-project.org/) to calculate historic (1950–2012) wildfire characteristics (i.e., number of fires, average fire area [km^2^], area burned annually [km^2^]) from the Bureau of Land Management-Alaska Fire Services and the Yukon Territorial Government's burn perimeter database records (data available at http://fire.ak.blm.gov and http://www.geomaticsyukon.ca/data/datasets) for our modeling domain. These databases contain fire perimeters dating back to 1917 and 1946, respectively.

### Modeling platform

We used ALFRESCO [1], [15], [45] to simulate the change in winter caribou habitat (2010–2100) in response to climate projections and the resulting fire regime. ALFRESCO is a spatially-explicit, stochastic landscape succession model for sub-arctic and boreal vegetation types that operates at a 1-km resolution and an annual time-step to model the interactions between fire, a changing climate, and 4 vegetation types (white spruce, black spruce, deciduous, and tundra) in Alaska. For this research, the model used downscaled scenarios of temperature and precipitation from each GCM given the A1B greenhouse gas emission scenario (see below for details). Wildfires were randomly “ignited” using a cellular automata approach based on a linear regression model developed by Duffy et al. [3]. Cell ignition and fire spread (i.e., flammability) is a function of climate, vegetation state, and time since last fire. The ignition of any given cell is determined by comparing a randomly generated number against the flammability coefficient of that cell. The flammability coefficient allows for changes in flammability that occur through succession (i.e., fuel build up). Following a wildfire in ALFRESCO, general successional trajectories were as follows: burned spruce forest (white or black) transitioned into early successional deciduous forest, and burned deciduous forest and tundra self replaces. Vegetation transition times differed probabilistically between climax black and white spruce trajectories [11]. Transitional times were modeled probabilistically to represent early successional (i.e., recolonization) deciduous vegetation following wildfires in spruce and deciduous forest and to determine the amount of time, in the absence of fire, until the climax spruce stage dominates the site again. Self replacement of deciduous forest can occur when repeated burning and/or climate conditions preclude transition to climax spruce. ALFRESCO incorporates the effects of fire severity on transition times using measurements of the area of the wildfire (i.e., fire size), complex topography, and vegetation type on flat landscapes [52]. We calibrated the relationship between climate and fire by comparing model output (e.g., fire regime, stand age structure) to the corresponding historical data [15], [53].

### Climate models

We used two downscaled Coupled Model Intercomparison Project 3 GCMs in the ALFRESCO simulations: Canadian Center for Climate Modeling Analysis Coupled Global Climate Model 3.1 (hereafter referred to as the “warm” GCM; more info at http://www.ec.gc.ca/ccmac-cccma/default.asp?lang=En&n=1299529F-1) and Max Planck Institute European Center-Hamburg 5 Model (hereafter referred to as the “hot” GCM; more info at http://www-pcmdi.llnl.gov/ipcc/model_documentation/ECHAM5_MPI-OM.htm). Global circulation models are mathematical representations of atmospheric and oceanic conditions and have the dual-purpose of simulating historical conditions based on known atmospheric values, such as greenhouse gas concentrations, and extrapolating those conditions to the future through the use of greenhouse gas emission scenarios. The GCMs we used represent the low and high limits of projected climate change of the five best performing GCMs for Alaska [54]. The GCMs were downscaled to 2-km resolution using the delta downscaling method by the Scenarios Network for Alaska and Arctic Planning (available at http://www.snap.uaf.edu/), and subsequently resampled to 1 km for input to the ALFRESCO model. We used the A1B emissions scenario [55], which assumed a mid-range of emissions in the future with a steady increase in carbon dioxide, however, recent climate and emission trends suggest that A1B may be conservative [56].

### Vegetation

The input vegetation map is a simplified version of the North American Land Change Monitoring System land cover map [57]. This land cover map was reclassified to meet the needs of the ALFRESCO model by collapsing classes into 5 groups: rock/ice, tundra, black spruce, white spruce, and deciduous. At the time of these analyses, there were no large-scale vegetation data available in a comparable format for the Northwest Territories, so we did not include this region in our simulations. This vegetation map was used as input in the ALFRESCO “spin up” phase of approximately 90 replicates running for 1,000 years to create a simulated landscape similar to current conditions with regards to burn characteristics (e.g., stand age), and vegetation distribution and composition [11], [15], [53].

### Analyses

We used the ALFRESCO simulations, driven by temperature and precipitation projections from each GCM, to analyze the number and size of fires and total area burned (mean ± SD), associated vegetation changes, and relative flammability of the landscape during 2010–2100 for each herds' winter range, as well as throughout the modeled spatial domain. For each GCM, ALFRESCO produced 90 simulation runs of annual series of spatial raster maps that depicted stand age and vegetation types over the 90 years of the simulation. Time (years) since fire from simulated historic (<2010) and projected (2010–2100) fire histories was used to determine age structures of vegetation communities on the landscape (i.e., pixels that burned in a previous year had an age of zero in the subsequent year). Forage lichen recovery generally occurs >60 years following fire [12], [26], [29], so stands of spruce or tundra in these age-classes were assumed to produce more lichen than younger stands (<60 years old), thus we classified older, lichen-producing stands as winter habitat for caribou. To find the most representative model run for each GCM, we compared the annual area burned from each run over the 90 simulated years with the median values for each year of all 90 runs and selected the run with the highest correlation coefficient (*r*) [53]. The annual representative runs were used to summarize fire characteristics (km^2^) and the amount of winter habitat (km^2^; measure of uncertainty  =  5^th^ and 95^th^ percentiles) by decade for each GCM.

We assessed the spatial distribution of relative flammability across the landscape for each GCM. Relative flammability was defined as the likelihood of a pixel to ignite throughout spatial and temporal domain of the simulations, thus we calculated the proportion of years among all the simulations (90 simulation runs x 90 years per simulation) that each individual pixel burned. From the resulting relative flammability maps for each GCM, we determined the top 20% of the modeling domain where fires occurred most frequently throughout our simulations, then calculated the percent of each herd's winter range that occurred within this highly flammable category. To identify areas within the domain with the largest relative differences in flammability between GCMs and, thereby, a proxy for the largest uncertainty in future fire characteristics, we subtracted the continuous relative flammability maps of the warm GCM from that of the hot GCM.

## Results

The annual representative runs from ALFRESCO (i.e., most correlated with median area burned of all repetitions across 90 years) that were used for subsequent analyses had correlation coefficients of 0.904 and 0.922 for the warm and hot GCMs, respectively. Throughout our simulations, fires were less numerous, smaller, and burned less area in tundra compared to spruce for both GCMs (Table 1). Fire characteristics followed general expectations for the warm and hot GCMs with the hot model projecting larger fires and more area burned. Due primarily to the increase in the size of fires, the average area of winter habitat that burned per decade was 64 and 25% higher in the hot versus warm GCM for tundra and spruce, respectively (Table 1).

**Table 1:** Simulated average (SD) annual fire characteristics by decade within lichen-producing vegetation classes (i.e., spruce and tundra >60 y) under a moderate emissions scenario (A1B) for two global circulation models [GCM; Canadian Center for Climate Modeling Analysis Coupled Global Climate Model 3.1 (Warm GCM) and Max Planck Institute European Center-Hamburg 5 Model (Hot GCM)] within the spatial domain of the modeling effort including the winter ranges of the Central Arctic and Porcupine caribou herds in northern Alaska and Yukon.

Decade	Warm GCM							Hot GCM						
	Tundra				Spruce			Tundra				Spruce		
	Number of Fires	Fire Size (km^2^)	Area Burned (km^2^)		Number of Fires	Fire Size (km^2^)	Area Burned (km^2^)	Number of Fires	Fire Size (km^2^)	Area Burned (km^2^)		Number of Fires	Fire Size (km^2^)	Area Burned (km^2^)
2010s	8.2 (4.2)	5.8 (1.8)	50 (27.7)		21.7 (4.9)	52.6 (30.4)	1219 (745.9)	10.4 (6.1)	26.7 (55.8)	506 (1186.2)		22.8 (4.8)	68.9 (86.8)	1794 (2398.6)
2020s	5.2 (1.8)	3.9 (1.8)	21 (14.2)		20.9 (4.7)	30.2 (16.5)	614 (296.5)	9.6 (6.3)	7.0 (7.4)	104 (203.4)		22 (5.9)	56.9 (54.2)	1449 (1692.2)
2030s	8.1 (3.4)	9 (6.9)	91 (104.1)		21.8 (4.3)	60.8 (44.7)	1385 (1152.6)	9.4 (3.4)	17.8 (23.9)	205 (292.5)		25.1 (3.6)	85 (71.8)	2140 (1930.5)
2040s	7.7 (4.6)	7.2 (5.4)	64 (62.5)		24 (5.4)	55.7 (36.4)	1426 (1020.6)	9.1 (4.7)	14.3 (10.8)	151 (165.2)		24 (7.8)	64 (40.8)	1781 (1401.1)
2050s	8.9 (4)	8.5 (5.1)	86 (74.4)		26.3 (4.9)	65.4 (34)	1719 (770.7)	9.1 (5.2)	11.9 (9.4)	150 (172.1)		21.4 (6.1)	79.4 (48.9)	1853 (1434.9)
2060s	9.8 (3.2)	7 (3.1)	69 (31.7)		24.3 (5.5)	43.5 (21.5)	1070 (587.4)	9.8 (3.7)	31.8 (56.2)	431 (924.2)		21.4 (5.9)	67.1 (44.1)	1633 (1516)
2070s	8.8 (2.7)	12.1 (11.9)	119 (150.3)		24.6 (5.9)	60.5 (47.3)	1649 (1471.3)	8.3 (3.8)	13.4 (12.5)	146 (190.3)		23.2 (5)	64 (63.9)	1616 (1744.4)
2080s	9.1 (3.2)	9.8 (6.9)	86 (52.5)		23.3 (3.8)	49.2 (24.6)	1167 (603.2)	8.6 (4.6)	31.4 (52.2)	460 (1021.8)		23 (6.3)	62.8 (37.6)	1589 (1511.3)
2090s	11.1 (4.1)	18.6 (15)	230 (204.1)		23.3 (4.4)	89.8 (39.5)	2025 (724.9)	11.4 (4.3)	55.3 (60.1)	689 (750)		23.3 (5.6)	93.5 (49.8)	2240 (1388.7)

**Figure 2:**
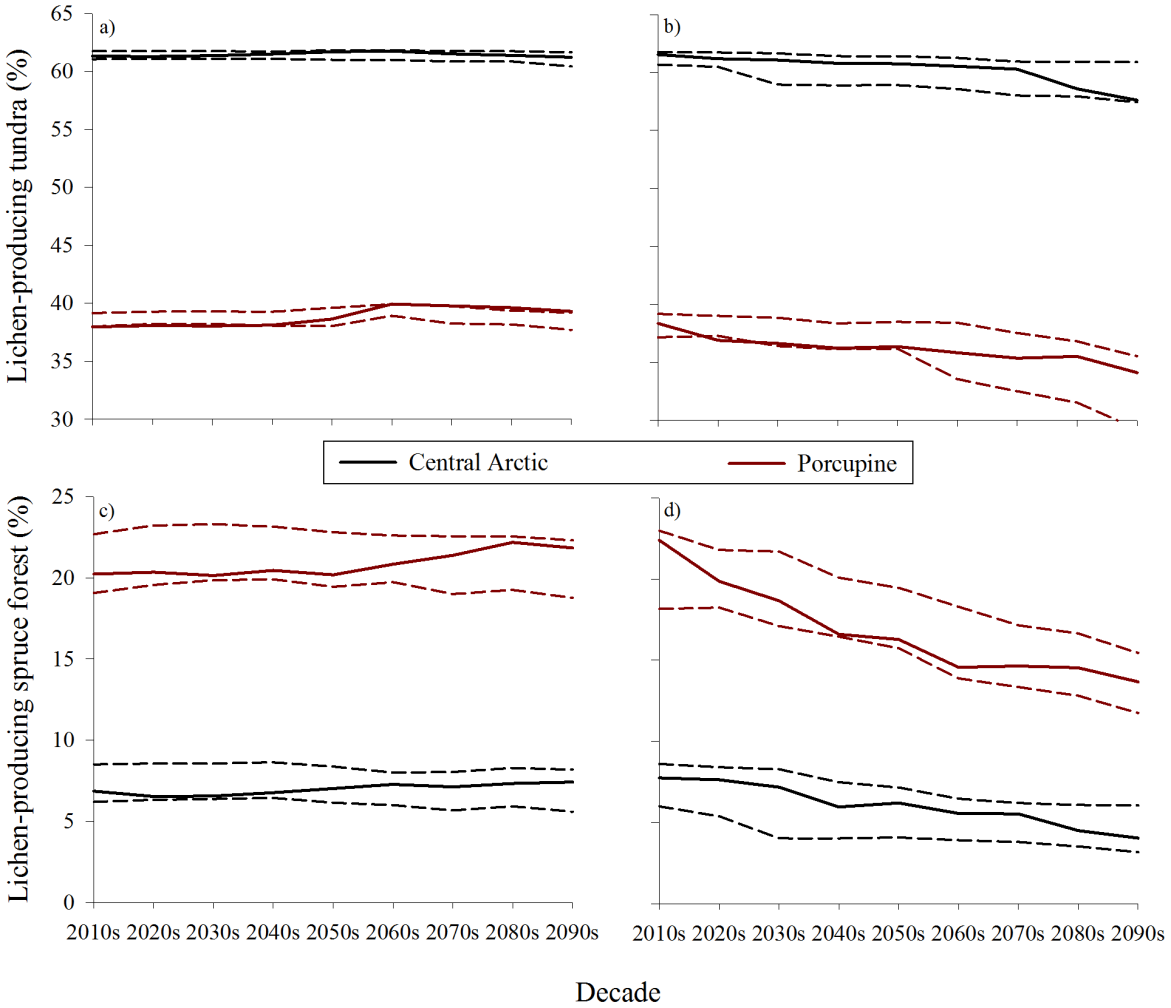
Simulated proportion of the winter ranges of the Central Arctic and Porcupine caribou herds in northern Alaska and Yukon that were covered in lichen-producing (>60 y) tundra (a, b) and spruce forest (c, d) under a moderate emissions scenario (A1B) for the warm [Canadian Center for Climate Modeling Analysis Coupled Global Climate Model 3.1 (a, c)] and hot [Max Planck Institute European Center-Hamburg 5 Model (b, d)] global circulation models, Alaska and Yukon. Representative runs are denoted by solid lines and the 5^th^ and 95^th^ percentiles are denoted by dashed lines.

The projected changes in the amount of winter habitat within each herd's range differed by vegetation type and GCM. For the Central Arctic herd, the percent of winter range that was winter habitat changed little through the nine decade simulation for the warm GCM, but decreased under the hot GCM (Fig. 2). Under the warm GCM, the extent of winter habitat was essentially the same through 2100, increasing <1% (41,009 km^2^ in 2010s versus 41,300 km^2^ in 2090s) with little change in composition of tundra and spruce communities (89.2 and 88.9% tundra, respectively). Conversely, under the hot GCM, winter habitat decreased by 11% throughout the same time period (41,692 to 37,092 km^2^) with a shift to less old-aged spruce habitats (11.2 and 6.5% spruce in 2010s and 2090s, respectively). For the Porcupine herd, results across the 90 years for the two GCMs differed more widely, with a modest increase (+5%; 102,710 to 107, 909 km^2^) in the availability of winter habitat under the warm GCM, compared to marked declines (−21%; 107,224 to 84,353 km^2^) for the hot GCM (Fig. 2). Similar to the Central Arctic herd's range, the composition of winter habitat within the Porcupine herd's winter range changed little under the warm GCM (64.3% tundra in 2010s and 2090s, respectively), but for the hot GCM, the proportion comprised of spruce habitats declined markedly (36.8 to 28.6%).

**Figure 3:**
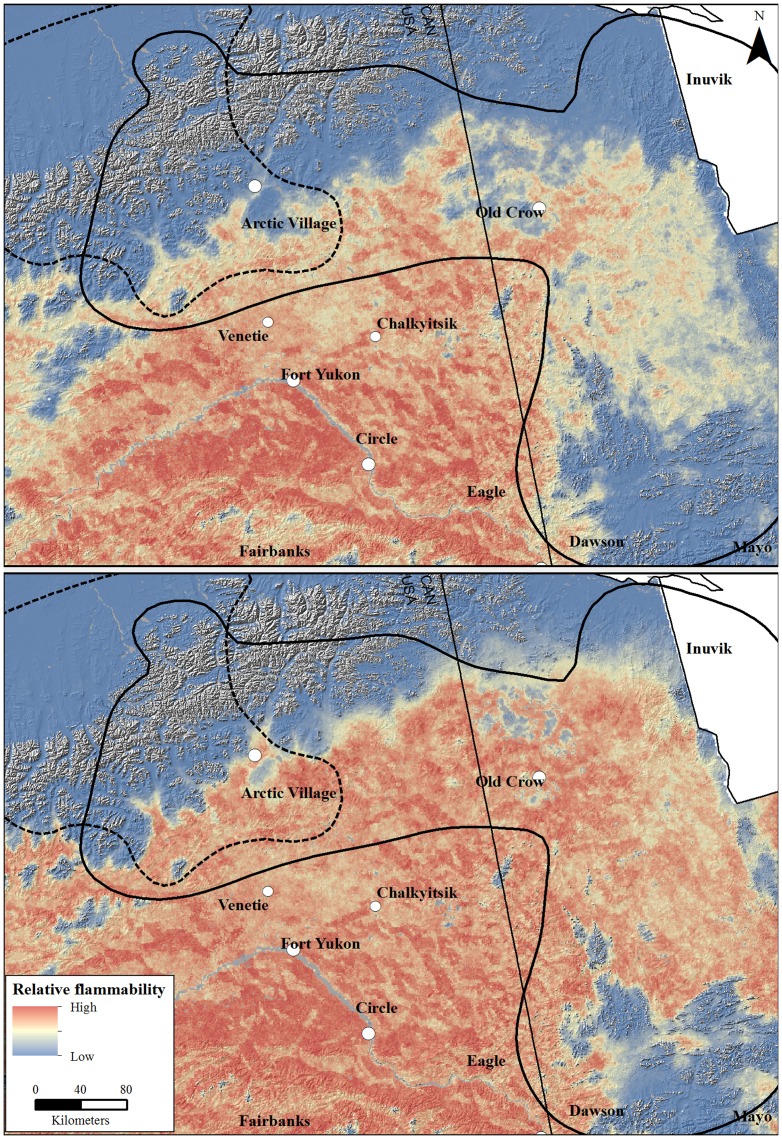
Relative flammability under a moderate emissions scenario (A1B) for the warm [Canadian Center for Climate Modeling Analysis Coupled Global Climate Model 3.1 (top panel)] and hot [Max Planck Institute European Center-Hamburg 5 Model (bottom panel)] global circulation models in the winter ranges of the Central Arctic and Porcupine caribou herds, Alaska and Yukon, 2010–2100.

**Figure 4:**
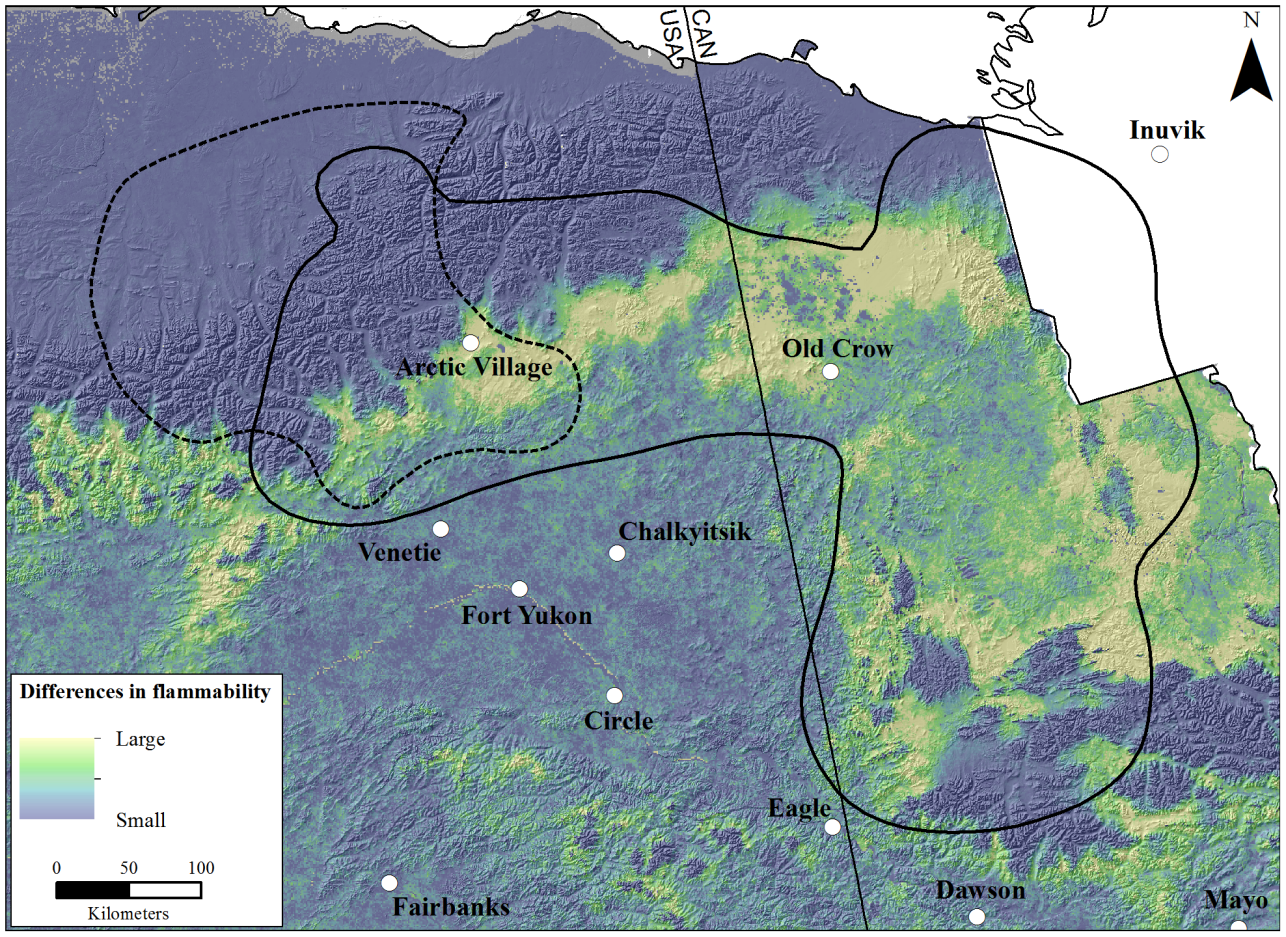
Differences in relative flammability of vegetation between the warm (Canadian Center for Climate Modeling Analysis Coupled Global Climate Model 3.1) and hot (Max Planck Institute European Center-Hamburg 5 Model) global circulation models under a moderate emissions scenario (A1B) in the winter ranges of the Central Arctic and Porcupine caribou herds, Alaska and Yukon, 2010–2100.

In assessing the capacity of areas to burn during the 90-year simulations for both GCMs, the Porcupine herd had a larger portion of its winter range within the highest relative flammability category (Fig. 3). For the warm GCM, 0.9 and 3.5% of the Central Arctic and Porcupine herd's ranges, respectively, had the highest occurrence of fires. The amount of highly flammable area increased approximately 2–4 times for both herds under the hot GCM (Central Arctic  = 3.4% and Porcupine  = 8.9%). In examining the spatial distribution of the differences in the relative flammability between GCMs, most of the uncertainty between simulations was located on the southern slope of the Brooks Range in Alaska as well as a large portion of the simulation domain in the northern interior Yukon (Fig. 4). Conversely, two prominent regions were consistent in flammability between the GCM simulations: the north slope of the Brooks Range had low relative flammability while the Yukon Flats in Alaska, including the communities of Venetie, Chalkyitsik, and Fort Yukon, had a high relative flammability (Fig. 4).

## Discussion

The projected influences of climate changes to fire regimes in northern ecosystems yielded insights into the potential availability of winter habitat for the Central Arctic and Porcupine caribou herds, with implications for other migratory tundra caribou populations throughout the 21^st^ century. The projected availability of winter habitat and relative flammabilities depended on the GCM and coarse vegetation type within the each herd's winter range (Figs. 2 and 3). Specifically, under the hot GCM we projected declines in winter habitat for both herds with larger decreases for the Porcupine herd that wintered primarily in the boreal forest (Fig. 2). If flammability of the Arctic tundra biome does indeed increase due to vegetation changes, drying, and increase in ignition agents [6], [11], [58], [59], losses of winter habitat for migratory tundra caribou may be amplified. Herd-specific simulations, however, will help to establish regionally appropriate climate-change projections for migratory tundra caribou as well as the repercussions to the communities that rely on these caribou as a subsistence resource [60].

Given current greenhouse gas emissions and climate trajectories, as well as the simplification of the successional pathways and associated flammability of tundra, our projections of winter habitat for these caribou herds were conservative. Since IPCC's fourth assessment, greenhouse gas emissions have exceeded expectations and considering recent trends, the A1B emissions scenario is likely an underestimate of what actual greenhouse gas emissions will be for the remainder of the century [19], [56]. Temperature projections in particular are most sensitive to greenhouse gas emissions scenarios [19], therefore future temperature regimes in Alaska may more closely align with the hot GCM. Coupled with a moderate emissions scenario, these two GCMs identified areas of uncertainty between scenarios in flammability (Fig. 4) and established a range of possible outcomes for caribou that included increases of 0.7 and 5% (warm GCMs) and decreases of 11 and 21% (hot GCM) of winter habitat for the Central Arctic and Porcupine herds, respectively (Table 1, Fig. 2). However unlikely, if greenhouse gas emissions decrease and (or) temperature projections align with the warm GCM, than this work provides a relevant alternative regarding the availability of winter habitat for these two herds.

Accounting for spatial variation in ignition agents and the capacity of a pixel to burn and the actual successional pathways of tundra vegetation will likely increase projections of the area burned [11], and thereby magnify the projected losses of winter habitat for migratory tundra caribou. In the current version of ALFRESCO, all types of tundra habitat, such as alpine, graminoid, shrub, or wetland tundra, are treated the same with regards to flammability and successional pathways (i.e., low flammability and tundra transitions to tundra after a fire) [11], [15]. However, flammability and successional pathways following a tundra fire appear to be related to initial tundra type, geography, local isotherms, fire severity, juxtaposition of seed sources, and colonization of shrubby species [20], [39], [61]. For example, during periods of warming, Paleo-records suggest birch-dominated shrub tundra burned as frequently as modern day boreal forest [20], [39]. Thus, expansion of shrub-dominated tundra [20], as is being observed on portions of the North Slope of Alaska [62], coupled with warmer temperatures throughout the growing season may increase the flammability of vegetation in areas where fires have, until recently, been rare [6]. In an attempt to deal with this spatial variation in flammability within tundra vegetation in northwestern Alaska, previous work altered the flammability coefficients in ALFRESCO by physiographic region [11]. Fire return intervals in that region of Alaska (approx. 140–240 yrs) [58] are not indicative of tundra vegetation on the North Slope of Alaska where fire return intervals may exceed 1,000s years [6], [20]. The fire regime in western Alaska is more similar to fire regimes of lowland tundra interspersed amongst spruce forest that is common throughout the winter range of the Porcupine caribou herd [23]. Regardless, any modification of tundra flammability would have decreased the fire return interval and increased the amount of area burned thereby amplifying the magnitude of the trends we simulated.

Despite the potential for shifting fire dynamics to influence the lichen-producing tundra, the projected availability of winter habitat for these caribou herds was driven by climate-fire dynamics in the boreal forest. Fire greatly influences ecosystem structure in the boreal forest by influencing successional trajectories [34]–[36]. The projected increases in temperature coupled with extended growing seasons, increased fuel buildup, and drying will reduce the mean stand age and increase the prevalence of deciduous stands [8]. Spruce forest comprised a relatively small portion of the Central Arctic herd's range (Fig. 2), and correspondingly, this herd incurred a smaller loss of winter habitat (∼11%) under the hot GCM. Although the simulated effects to the Central Arctic herd appeared relatively minor under the hot GCM, if the recent heavy use of the forested southern slope of the Brooks Range in winter is indicative of a longer term trend [47], then any losses of these old-growth spruce forests may become more influential to the distribution of this herd in winter. Alternatively, old-growth spruce forest was more abundant within the winter range of the Porcupine caribou herd (Fig. 2). Comparable to simulations in western Alaska [11], we project this herd will lose 21% of winter habitat to fire by the end of the century. The majority (67%) of this loss was driven by increased flammability in spruce forests in the Yukon (Fig. 3). Thus, managers there will likely contend with relatively significant changes in the fire regime and subsequent reductions in the availability of lichen-producing vegetation. Based on the expected shifts in fire regime throughout the boreal forest [8] and the predominantly boreal-wintering behavior of migratory tundra caribou in North America, these projections for the Porcupine herd may be indicative of fire regime shifts influencing other continental caribou populations.

As noted throughout fire-caribou literature over the past 50 years, the effects of fire-driven habitat changes to caribou population dynamics is uncertain. The linkage between the availability of winter habitat, shifts in distribution, and changes in abundance of herds is difficult to establish for populations that range over large areas and demonstrate remarkable demographic plasticity in spite of austere and variable conditions. At the heart of this complexity and uncertainty are the numerous intra- and inter-annual factors that affect caribou populations and, to some degree, are influenced by climate changes. These include the influence of snow conditions on forage availability and energetic costs of movement and foraging [63], capacity to shift seasonal ranges to account for changes in range quality [14], [64], vegetative shifts that may increase apparent competition [11], intra-specific competition [65], top-down effects of herbivory on vegetation communities [66], [67], expansion of the growing season and increases in primary productivity that may confer nutritional benefits to reproductive females [68], [69], influence of tundra fires on the quality of summer habitats [in sensu 70, 71], insect harassment in the summer [72], and anthropogenic influences [73]–[76]. Caribou populations can and do shift their distributions to minimize any or all of the above factors [64], [77], [78]. However, available wintering habitat is continuously occupied by migratory tundra caribou herds from the Bering Sea to the Hudson Bay and is limited to the north by the Arctic Ocean [13], [79]. Climate-fire-winter habitat dynamics will most certainly affect the availability of winter habitats for these herds as well, but to varying degrees. Thus, the capacity for a herd to shift its range to accommodate these changes will vary with the availability of winter habitat in the adjacent herds' ranges. Despite these complexities, it is relatively clear: caribou will need to alter their distribution to avoid recently burned areas in winter due to reduced lichen presence [11], [12], [14], [29], [38], [80].

Although the population-level effects may be unclear, the potential changes in caribou distribution will most certainly affect human communities that have a cultural and nutritional reliance on caribou. Unlike caribou herds, communities have limited resilience to large shifts in availability of food resources. For example, four indigenous communities occur in a region that, regardless of GCM, had a relatively high flammability (Figs. 3 and 4). One community (Old Crow, Yukon Territory) is within the traditional winter range of the Porcupine herd, while hunters from three villages (Fort Yukon, Venetie, and Chalkyitsik, Alaska) travel north each year to harvest animals from this herd. With caribou avoiding or shifting migrations away from recent burns, harvest opportunities are impacted immediately by wildfire and the effects could last for two generations of hunters [81], [82]. Also, wildfire indirectly affects hunting opportunities by impeding hunter travel across the landscape [7], [60], [83]. Based on simulated relative flammabilities, projected increases in fire sizes under the hot GCM, and historic distribution of the Porcupine caribou herd (Figs. 3 and 4), it is unlikely that caribou will become more accessible to these aboriginal communities in winter.

Projecting the influences of climate changes to wildlife populations is a necessary but daunting task fraught with numerous ecological, climatic, and technical complexities, uncertainties, and assumptions [84]–[86]. As noted in other efforts using ALFRESCO, this work was not insulated from these challenges [11], [15], however, certain aspects of the life-history characteristics of migratory tundra caribou and their habitats facilitated the projection climate-fire induced changes to the availability of winter habitat. These characteristics included the clear roles that vegetation, temperature, and precipitation have in structuring fire regimes, the strong influence of fire on successional pathways in northern systems, the specific stand characteristics that facilitate lichen colonization and growth in boreal and arctic systems, and the important role of lichens in the winter diets of caribou. Projected warming and greenhouse gas emission trends will indeed alter fire dynamics and shift vegetative composition and age structure to lower the availability of winter habitat to migratory tundra caribou [11], [15]. Yet, linking these projected losses of winter habitat with changes in abundance of caribou, and rectifying the apparent opposing influences of other climate-driven changes to winter habitats [negative: this study, 11] and throughout the growing season [69] remain important issues in elucidating climate-induced effects to caribou populations and the communities that depend on them.
