# Correction: Climate-Driven Effects of Fire on Winter Habitat for Caribou in the Alaskan-Yukon Arctic

**DOI:** 10.1371/journal.pone.0112584

**Published:** 2014-10-30

**Authors:** The PLOS ONE Staff

Figure 3 is incorrect. The authors have provided a corrected version here.

**Figure 3:**
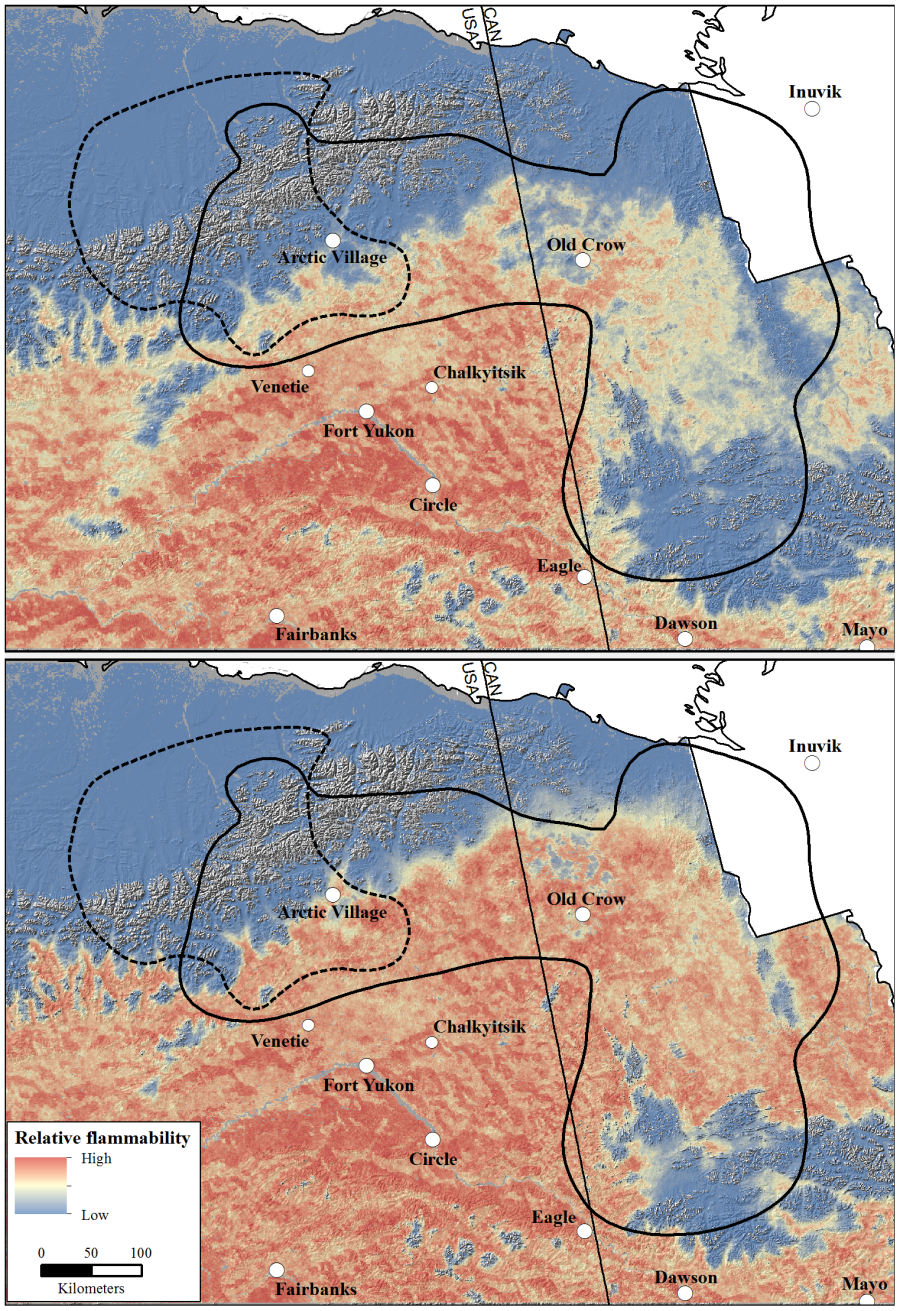
Relative flammability under a moderate emissions scenario (A1B) for the warm [Canadian Center for Climate Modeling Analysis Coupled Global Climate Model 3.1 (top panel)] and hot [Max Planck Institute European Center-Hamburg 5 Model (bottom panel)] global circulation models in the winter ranges of the Central Arctic and Porcupine caribou herds, Alaska and Yukon, 2010–2100.

## References

[1] Gustine DD, Brinkman TJ, Lindgren MA, Schmidt JI, Rupp TS, et al (2014) Climate-Driven Effects of Fire on Winter Habitat for Caribou in the Alaskan-Yukon Arctic. PLoS ONE 9(7): e100588 doi:10.1371/journal.pone.0100588 24991804 10.1371/journal.pone.0100588 PMC4081032

